# Clinical proteomics of enervated neurons

**DOI:** 10.1186/s12014-016-9112-2

**Published:** 2016-05-05

**Authors:** Mohor Biplab Sengupta, Arunabha Chakrabarti, Suparna Saha, Debashis Mukhopadhyay

**Affiliations:** Biophysics and Structural Genomics Division, Saha Institute of Nuclear Physics, 1/AF Bidhannagar, Kolkata, West Bengal 700064 India

**Keywords:** Neurodegeneration, Proteomics, Alzheimer’s disease, Spinal cord injury, Neuromyelitis optica

## Abstract

The dynamic field of neurosciences entails ever increasing search for molecular mechanisms of disease states, especially in the domain of neurodegenerative disorders. The previous century heralded the techniques in proteomics when indexing of the human proteomes relating to various disease conditions became important. Early stage research in certain diseases or pathological conditions requires a more holistic approach of first discovering the proteins of interest for the condition. Despite its limitations, proteomics is one of the most powerful techniques available to us today to dissect the molecular scenario in a particular disease situation. In this review we will discuss about the current clinical research in neurodegenerative disorders that employ proteomics techniques. We will specifically focus on our understanding of Alzheimer’s disease, traumatic spinal cord injury and neuromyelitis optica. Discussions will include ongoing worldwide research in these areas, research in India and specifically our laboratory in these domains of neurodegenerative conditions.

## Background

The most polarized cells of the human body, neurons, are a specialized type with respect to their functional properties. Development and function of neurons are closely linked to the bidirectional transport of molecules from the synaptic end to the cell body. This very synaptic signal, which when disrupted, causes the dysfunction of neuronal activities. Disruption in axonal transport is the cause of several neurodegenerative disorders [1, 2]. In the realm of peripheral neuron injury, retrograde transport of molecules from the site of injury to the cell body of a peripheral neuron primes the latter to regenerate [3, 4]. This phenomenon is absent in the central nervous system (CNS), with the consequence of regeneration after CNS injury being elusive even with years of research. Partly because of the large distance separating the axon end from the cell body, many molecular events after a trauma or a neuronal disease occur without any transcriptional manifestations [5]. Local proteolysis, protein synthesis and post translational modifications are the key to understanding axonal events after an assault or a disorder of the neuron [5]. Proteomics approaches have therefore come to the limelight in recent times. In this review, we will discuss the contributions of our group from this perspective and also the prospective ideas in three neurological degenerative situations, namely Alzheimer’s disease (AD), traumatic spinal cord injury (SCI) and neuromyelitis optica (NMO) and explore the advances in understanding these pathological processes using proteomics approaches.

### Ethics statement

The data provided in the review was collected by a joint collaborative study of SINP and NRSMC&H, Kolkata, India, after it was approved by Institutional Ethical Committees. An informed written consent was obtained from the subjects as per Helsinki Declaration, 2013.

## Clinical proteomics in AD

During the past few years Mass spectrometry (MS) based proteomics tools have been used extensively to study AD-related proteome changes in blood (plasma and serum), cerebrospinal fluid (CSF) samples and in postmortem brain tissues [6]. Since the pathological processes of AD start decades before the first symptoms appear, the objective of all AD proteomics studies have been to identify precisely the early diagnostic and prognostic biomarkers. Here we review reports that have used diverse samples including blood, CSF, brain tissues and also discuss different aspects of proteome status like posttranslational modifications (PTMs), redox proteomics and interaction proteomics.

Blood and CSF proteomics studies are being done for more than a decade to identify AD-related biomarkers, of which the most widely researched one is the peptide Amyloid β (Aβ). Utility of Aβ as a predictor of dementia and AD is well established and it is evident that lower Aβ_42_:Aβ_40_ ratios are mainly associated with the disease [7]. In 2007, a plasma proteomic study in AD patients identified six potential plasma biomarkers using 2D-GE and LC/MS/MS [8]. Some of them, for example α-1 antitrypsin, could be validated for its higher expression level in plasma of AD patients using ELISA. Apolipoprotein J was found to be in lower abundance in plasma of AD patients in an isoform-specific manner. This observation could only be achieved through 2-DE but could not be validated through biochemical methods like ELISA or Western blot. Recently, a large scale replication study was conducted for 94 proteins out of 163 potential candidate biomarkers found in 21 published blood proteomics studies. 9 were found to be associated with AD-related phenotypes [9]. It was concluded that there are replicable changes in proteomic expressions in blood of AD patients that can be identified by different studies with some consistency.

The rationale of studying plasma and CSF biomarkers for AD has been explicitly reviewed [10] and the diagnostic performance of the core CSF biomarkers, namely total tau, phosphorylated tau and Aβ-42 has been discussed. But studying these traditional biomarkers is not sufficient to identify preclinical AD. Study of differential proteome from whole tissue/body fluids in the disease model compared to that of the normal control is of immense importance in order to identify key, novel protein candidates for the disease. Recently, combination of Aβ, tau and p-tau along with several novel biomarkers in CSF have been used extensively for diagnostic confirmation of AD [11]. It is realized that co-development of biomarkers for early diagnosis and novel therapeutic approaches, together could be the way to go.

A recent review has extensively discussed the features of neurodegeneration proteomics and its importance in relation to PTM [12]. Evaluation of functional alterations in proteins due to PTMs can be achieved by means of a battery of proteomic tools. For the elucidation of pathogenic mechanisms, study of PTMs in AD is of immense importance and would become the future of proteomics research to understand the disease process. Very recently, the CSF glycoproteome was studied as a fingerprint of the brain glycoproteome of AD patients [13] and it is concluded that CSF N-glycome analysis may provide reliable biomarkers for early diagnosis of the disease. In many studies, proteins from CSF of AD patients are shown to have differential isoforms in terms of glycosylation [14], S-nitrosylation [15] and histone protein modifications [16] in different tissue sections of the AD brain. Recently quantitative phosphoproteomics studies in the frontal cortex [17] and hippocampi [18] of AD brains have deciphered the deregulation of biological pathways in AD due to alteration in the PTM status. A more recent study deals with changes in protein phosphorylation status in the inferior parietal lobule of subjects with different stages of the disease (AD, Mild Cognitive Impairment, MCI, Pre-Clinical Alzheimer’s Disease, PCAD) and control brain [19]. Phosphorylation status of nineteen proteins is found to be different in these stages of the disease. These proteins are involved in energy metabolism, neuronal plasticity, signal transduction and oxidative stress response. The same group has studied and discussed the effects of Aβ-induced oxidative stress, its redox proteomics and its importance in disease pathology and progression [20, 21]. Also, selectively oxidized proteins namely, creatine kinase BB, glutamine synthase, ubiquitin carboxy-terminal hydrolase L-1, alpha-enolase, triose phosphate isomerase and neuropolypeptide h3 in human samples were identified and can be studied in future to understand the AD pathogenesis better [22, 23]. Redox proteomics analysis has been conducted to identify specific carbonylated proteins (for example, alpha-enolase, glutamine synthetase, protein disulfide isomerase A3 etc.) in the hippocampus at the very early stage in AD mouse model [24].

A recent study with micro dissected neurons from the temporal cortex of AD brain has identified more than 400 proteins using LC–MS analysis [25]. Several recent studies have dealt with targeted and localized quantitative proteomics in presynaptic and postsynaptic tissue samples in AD and have identified a large number of proteins relevant to the disease process [26–28]. All these studies aim at identifying novel candidates or imprints for early diagnosis of a neurodegenerative condition like AD.

## AD clinical proteomics in India: Interaction proteomics with focus on APP intracellular domain (AICD)

There have not been many studies in the domain of Alzheimer’s disease research employing proteomics approach. Although there are extensive reviews discussing employment of proteomics for biomarker discovery in neuropsychiatric disorders [29] and of the relation of AD and type-2 diabetes [30], only one clinical proteomics study with AD CSF samples has been conducted [31]. This study has found Apolipoprotein E, Apolipoprotein J, Hemopexin, Complement factor b and complement C4b to be differentially abundant in AD versus control CSF samples.

Our laboratory focuses on interaction proteomics of amyloid precursor protein (APP) intracellular domain (AICD). AICD generated by post γ-secretase cleavage of APP has been an important molecule of the AD pathway and has drawn the focus of many researchers [32–35]. In the dephosphorylated state, it acts as a transcriptional transactivator to regulate the expression of several genes including GSK3β [36]. Once phosphorylated, it interacts with a plethora of other cellular adaptors, heavily influencing the cellular machinery. In an attempt to identify the intracellular interacting partners of phosphorylated AICD, we found twenty novel AICD interacting partners from mouse neuroblastoma cells [37]. Molecular functions of several of these interactors, for example ApoA4, GAB2, HSPA8, vimentin etc., could be correlated to AD, revalidating that AICD and its interactions have implications towards AD pathophysiology. In a different study, we could identify AICD interacting proteins from CSF of AD patients and also compare the differential expressions of these interactors with respect to that of non-demented control patients [38]. Keeping the need for identifying the indirect candidates involved besides the direct ones for in-depth exploration of the molecular networks of the disease, we transfected AICD in mouse and human neuroblastoma cell lines and identified differential expressions of proteins belonging to diverse molecular pathways [39], for example, ER-stress (GRP78), structural remodeling (vimentin) and general stress (HSP90β, HSPA8).

## Clinical proteomics in spinal cord injury

Acute traumatic injury of the spinal cord resulting in partial or total deficits in sensory and/or motor functions is widely prevalent worldwide and leads to considerable decline in the quality of life [40]. Though relative annual incidences of SCI vary with the geographical region and the time period of the study [41], studies show that global average incidence is highest in Asia [40] with 43.8 persons per million people afflicted. Traffic accidents [42], fall from height [43] and violence [44] constitute some of the major reasons for SCI.

Autonomic dysfunctions that follow SCI set the major decline in the quality of life of afflicted persons. An injury above the C3 level in the vertebral column may lead to immediate cardiac arrest necessitating assisted respiration [45]. While the autonomic deregulations may last for a period of weeks after an injury, grossly the pathophysiology of SCI is divided into primary and secondary injuries. The initial trauma to the spinal cord is called primary injury and it is a prognostic indicator of SCI [46]. Following the primary injury, secondary injury sets in [47] and may last from months to years. This phase witnesses a plethora of degenerative phenotypes attributed to multitudes of molecular responses to injury [48].

Therefore, in spinal cord injury research, it is very important to get a clear picture of the molecular anatomy at the vicinity of the injury. On the other hand, for the peripheral nerve injuries, which comprise a greater subset of neuronal injuries, study of molecular parameters promoting axonal regeneration is of paramount importance [49]. Axon forms an important tissue to analyse changes following an injury. This is because of a temporal sequence of three events promoting neuron regeneration: discharge of axonal potentials induced by injury, interrupted normal supply of retrogradely transported target-derived factors, and retrograde injury signals travelling from the injury site back to the cell body, also called positive injury signals [50]. Here, the necessity of biomarker analysis is born. Especially, proteomics approaches are preferred to those of genomics in the domain of axonal regeneration and degeneration because changes in axons often occur without any transcriptional events in the cell body, following a pathological condition [5]. Moreover, a large number of PTMs to proteins accompany axonal degeneration and regeneration, which has been rightfully termed as a ‘postgenomics’ problem. A series of elegant work from the group of Perlson et al., beginning with proteomics approach, showed that vimentin is translated at the site of axonal injury [51], it is retrogradely transported from the site of injury to the cell body on dynein motor [52] and binds with ERK preventing its dephosphorylation [53].

## Clinical proteomics tools to discover molecular interplay post SCI

Application of proteomics in biomarker analysis in traumatic SCI has found inflammatory cytokines at elevated levels and documented the elevation of tau, S100beta, GFAP and MCP-1 in a severity dependent fashion [54]. Commonly deregulated proteins as fished out by proteomics studies, include plasma proteins, HSPs, glycolytic enzymes, antioxidants and proteins participating in DNA damage and repair, protein degradation, cell signaling and structural proteins [55, 56]. Transgelin and protein S100-A11 were found to be biomarkers of bladder healing in the secondary injury phase of rats [57]. An early study with insoluble segments of injured rat spinal cord using 2-DE followed by MALDI-TOF/TOF found decreased abundance of pyruvate dehydrogenase beta, aconitase 2, fumarate hydratase 1, and ATP synthase subunit 6, which can lead to ATP depletion. On the other hand, antioxidant proteins such as catalase and PRDX-1 were decreased [58]. Implication of antioxidants were further shown with the aid of 2-DE where decreased abundance of catalase (CAT) and Mn-superoxide dismutase (Mn-SOD) were detected at the lesion centre 14 days post SCI in rats [59]. Additionally galectin-3, beta actin, actin regulatory protein (CAPG) and F-actin capping protein subunit beta (CAPZB) were found increased at similar time period post injury suggesting a decrease in antioxidant function and increase in growth inhibiting proteins post SCI. Treatment with acidic fibroblast growth factor (aFGF) down-regulated regeneration-blocking secondary phase proteins like S100beta, GFAP and the keratin sulphate proteoglycan, lumican, as demonstrated using proteomic approaches in rat SCI model [60]. Tyrosine 3-monooxigenase/Tryptophan 5-monooxigenase activation protein (YWHAZ), a hub of several signal transduction pathways, glutathione peroxidise 3, involved in detoxifying hydrogen peroxide and S100a8, which is zinc and calcium binding protein involved in immune response regulation, were found to be biomarkers of severity in rats at 24 h post injury [61]. Afjehi-Sadat et al. showed higher abundance of 14-3-3 epsilon protein, dynein light chain 1, and tubulin beta-5 chain and decreased abundance of adenylyl cyclase associated protein 1, dihydropyrimidinase-related protein 2, F-actin capping protein subunit beta, glyceraldehyde-3-phosphate dehydrogenase, stress-induced phosphoprotein 1 and transthyretin in injured tissue of SCI in rats. Free oxygen radical attack on proteins in SCI was indicated by PTM analysis [62].

Limitations of identifying a larger number of proteins using conventional 2-DE methods prompted researchers to use multiplex array proteomics to analyse low abundance proteins from CSF available in small volumes. A cytokine profiling from CSF of cervical SCI rats at 12 days post injury revealed MMP-8 to be an elevated biomarker [63]. Proteomics approaches have been employed to decipher the temporal changes of protein expression after SCI. This is particularly important because protein expression pattern is drastically disturbed following an injury and there is a prolonged persistence of irregularities in protein expression patterns thereafter. Zhu et al. found that 24 h post injury is the key time when the protein expression changes drastically in a rabbit SCI model [64].

Use of labeling with iTRAQ reagent coupled with proteomics approaches have derived significant results in the domain of SCI. LC–MS/MS with iTRAQ reagent labeling identified proteins involved in ubiquitination, endocytosis and exocytosis, energy metabolism, inflammatory response, oxidative stress, cytoskeletal disruption, and vascular damage as altered significantly at 24 h after SCI in rats [65]. Heat shock proteins were significantly differentially expressed in a rat model of SCI found in a study using iTRAQ and 2D LC–MS/MS [66] pointing to their potential role after SCI. 2-DE followed by nano ultra-high performance liquid chromatography-electrospray tandem mass spectrometry (NanoUPLC–ESI–MS/MS) identified proteins associated with apoptosis, nerve signal transduction and metabolism to be differentially regulated in rat SCI followed by treatment with basic fibroblast growth factor long circulation liposome (bFGF + LCL) [67].

## Proteomics of SCI in India

Currently, even though there are numerous studies being done on various, mostly rehabilitative [68], epidemiological [69], financial [70], clinical [71, 72] and surgical [73] aspects of SCI in India, use of proteomics approach to get into the molecular level has been done only in our laboratory.

We have been focusing on the molecular interplay occurring in the secondary phase of SCI, which essentially manifests as the various phenotypic dysfunctions associated with this stage. For this purpose we conducted a clinical sampling with 14 SCI patients at 1–8 days post injury and contrasted their cerebrospinal fluid (CSF) from complete and incomplete injury types. The injury severities were ascertained using the American Spinal Injury Association (ASIA) Impairment Scale (AIS) [74]. 2-DE followed by MALDI MS/MS was employed to identify CSF proteins from SCI patients and furthermore, 2D-DIGE was conducted to contrast the different AIS samples of CSF [56]. Forty-nine proteins were identified from CSF of SCI cases. Eight of them were differentially abundant (≥±1.5 fold) among AIS A (complete injury) and AIS C (incomplete injury) CSF samples. The status of the differentially abundant proteins among the AIS groups was further checked for CSF taken at 15–60 post injury from an additional 6 patients.

Application of bioinformatics tools to the identified proteins from SCI CSF yielded a protein–protein interaction network (PPIN) consisting of the identified proteins and their secondary interactors. From this network, interaction modules were created where protein members within a module interact more with one another than with proteins outside of the module. We adopted the Newman–Girvans modularization (NGM) algorithm [75–77]. Thus a modularised network was formed. The network was further enriched [78] to identify biological functions associated with the modules. This was done using GeneCodis3 [79].

Finally identifying the modules where the differentially abundant proteins were found, we zeroed in on the perturbed biological pathways post SCI at the secondary phase. The significantly perturbed pathways were mRNA metabolism, protein phosphorylation, iron transport, lipid and ATP catabolism, tRNA and rRNA transcription and DNA repair. We therefore identified some molecular pathways that lose their balance post SCI and we started off with identifying the entire proteome of SCI CSF and gradually narrowing down from there. The employment of proteomics tools was immensely useful in our case as we did not start with any particular protein or pathway in mind. This holistic approach with gradual focus on the important results is thus a preferred choice not only in the domain of biomarker discovery but also in a situation like ours, where a molecular dissection is being carried out.

## Neuromyelitis optica

Neuromyelitis optica (NMO) or Devic’s disease is an autoimmune inflammatory disease that affects the central nervous system, predominantly the optic nerves and spinal cord [80, 81]. Devic’s disease, because of its resemblance with multiple sclerosis was initially speculated to be a variant of the latter. But with advances in clinical and immunological studies, the existence of NMO as a distinct disease has been established [82, 83]. The clinical features associated with the disease include optic neuritis and transverse myelitis [84]. The disease can be either monophasic or relapsing [84]. In 2006 after the discovery of anti AQP4-IgG [85] the initially proposed diagnostic criteria for NMO was revised. The seropositivity of NMO autoantibody together with optic neuritis and longitudinal extensive transverse myelitis was then considered as the defining parameter for NMO [86].

As compared to other neurodegenerative disorders the global distribution of NMO is rather limited [87]. Its prevalence among Caucasians is low where the abundance of multiple sclerosis is high [88, 89]. Higher incidence of NMO has been reported among south Asian countries like India, Korea and the Philippines [90]. Demographic studies reveal that women are more predisposed to the disease than men [91, 92]; this is true even for the paediatric cases [93]. The disease mainly afflicts young adults with ages ranging mostly under 18 years although there have been some reports of late onset too [94, 95]. The administration of immunosuppressive drugs has been the major treatment for the disease [96].

## Advances in proteomic study of NMO

The presence of serum antibody NMO-IgG among the patients [85] was initially considered to be highly specific for disease confirmation but later it was seen that all patients were not NMO-IgG seropositive. Evidently it became crucial to look for other biochemical markers of the disease. Proteomic analysis which has been extensively employed in biomarker discovery for similar diseases like multiple sclerosis [97] was extended thereafter for the detailed study of NMO. The major study of NMO proteomics has been conducted in CSF, blood and urine. The insight into different CNS diseases is provided by CSF proteomics [98, 99]. CSF comparative proteomics with multiple sclerosis, NMO and normal patients by Jiang et al. led to identification of four proteins namely—Pre-Albumin (PA), Keratin1, transferrin and Keratin 9 [100]. PA had high expression levels among multiple sclerosis patients while Keratin 1 was significantly increased in NMO patients. Transferrin (Tf) is a crucial marker for blood–brain-barrier (BBB) damage [101] and its high levels in NMO are indicative of the damage caused to the blood–brain-barrier because of NMO pathogenesis. In a separate study by Bai et al., proteome analysis in the CSF of NMO patients in comparison to control group revealed the up regulation of Neurofilament, Haptoglobin, immunoglobulin kappa chain C region (IGKC) and immunoglobulin heavy chain gamma 3 (IGHG3) levels [102]. In the same group of patients there was downregulation in alpha-1β-glycoprotein (A1BG), fibrinogen gamma chain (FGG), apolipoprotein A-IV (ApoA-IV), apolipoprotein E (ApoE), transthyretin (TTR) and vitamin-D binding protein (DBP) levels. Among NMO patients the rise in IGKC and IGHG3 and the downregulation of ApoA-IV levels hints at the involvement of immunological mechanisms in disease progression. Neurofilament, a protein localised in the axon usually reflects the axonal health [103]. The expression of this protein in NMO poses it as a biomarker for the disease. In another serum proteomic study by Jiang et al., there was a two-fold elevation in the levels of haptoglobin in NMO patients in comparison to control as well as multiple sclerosis cases [104]. Nielson et al. revealed an increase in levels of immunoglobulins Ig-G3, Ig-K and Ig-L in the urine proteome of NMO subjects in comparison to healthy subjects [105]. The differential expression profiles that have been obtained using two dimensional (2D) gel electrophoresis in combination with mass spectrometry, in totality, projects several probable biomarkers that would guide in the diagnosis of NMO.

The futuristic approach of such proteomic profiling studies is to investigate the potential cellular players that are differentially expressed among the seropositive and seronegative groups of NMO patients. This would be extremely crucial both in terms of classification as well as treatment of these two different subgroups of NMO patients.

## Conclusions

The endeavor in our laboratory for the past 8 years has been centered on understanding mechanisms leading to nervous system trauma—be it a disease or an injury. Starting with Alzheimer’s disease, additional requirement of biomarkers aside from the most basic CSF ones, tau, phosphorylated tau and Aβ-42, prompted proteomics based research in this domain. The search identified several plasma and CSF biomarkers for AD. Further on, research from our laboratory has highlighted the interaction proteome of AICD in a cell based and clinical model. The follow-up studies dealing with candidate biomarkers identified through proteomics which are functionally linked to important biochemical pathways, are very crucial for the advancement of understanding the disease pathophysiology.

Coming on to spinal cord injury, the bulk of biomarker discovery has always employed proteomics methods. Since the lack of primary neuron regeneration beyond the injury point remains evasive, there is tremendous ongoing research in this area. Proteomics research in past decades have highlighted the role of cytokines in trauma pathology as well as those of plasma proteins, HSPs, glycolytic enzymes, antioxidants and DNA damage and repair proteins, protein degradation, cell signaling and structural proteins. Research from our laboratory has highlighted several perturbed molecular pathways post SCI, which include protein phosphorylation, DNA repair, mRNA metabolism, iron transport tRNA and rRNA transcription and lipid and ATP catabolism.

Neuromyelitis optica is a relatively less studied pathological condition when compared to the previous two and we have just started working with clinical samples. Anti AQP4-IgG was discovered in 2006 as a biomarker for NMO. A curious feature of this disease is that in several cases it has been observed that patients with NMO were not seropositive for the NMO-IgG. This prompted us to look for reliable candidate biomarkers and although studies have been limited so far, proteomics approaches look promising in this particular upcoming area of neurological disorder.

**Fig. 1:**
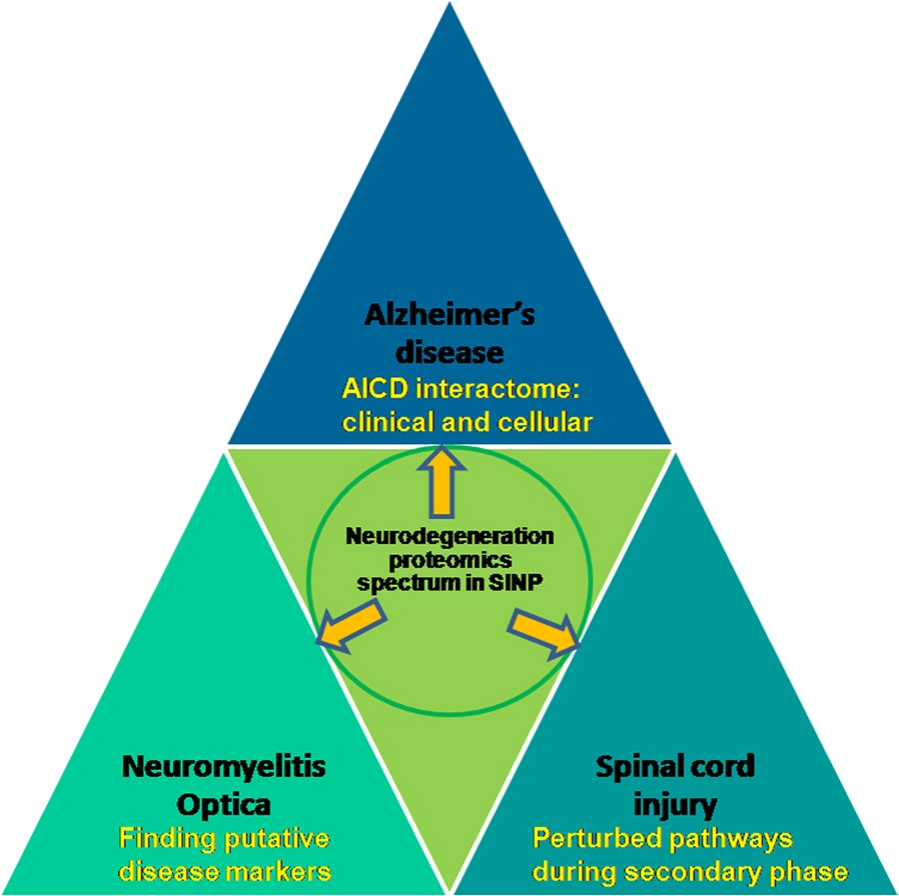
A representative diagram of the paradigm adopted by our laboratory in neurodegeneration research. Starting with Alzheimer’s disease, in proteomics domain, we found out the interacting partners of AICD in CSF and AICD-transfected human and mouse cell lines. Twenty novel AICD interactors were found in mouse neuroblastoma cells. The study was further followed up in human CSF, where differentially expressed AICD interactors were found out. Finally, differential expression of AICD interactors were studied in human and mouse neuroblastoma. Moving forward to spinal cord injury, we looked at differentially abundant proteins in the CSF of SCI patients with different severity grades of injury. An interaction network was created using the proteins found in the CSF of AIS grade A injury and modularization of the same revealed a number of perturbed pathways. Finding putative disease markers for the rather evasive successful marker hunting in neuromyelitis optica remains a future perspective

While working with nervous system we believed that proteomics approaches favor the initial ‘biomarker discovery’ stage of a research paradigm of a disease. It is particularly useful in diseases of the neurological disorders or injury as CSF and plasma form the common target sources of protein profile detection in the initial and also subsequent stages of identification of pathology mechanisms. Figure 1 represents the research strategies adopted by the Mukhopadhyay laboratory to study the three neuro-pathological conditions, AD, SCI and NMO.
